# Progress in the Research and Development of Anti-COVID-19 Drugs

**DOI:** 10.3389/fpubh.2020.00365

**Published:** 2020-07-07

**Authors:** Lianzhou Huang, Yuanqiu Chen, Ji Xiao, Weisheng Luo, Feng Li, Yuan Wang, Yiliang Wang, Yifei Wang

**Affiliations:** Guangzhou Jinan Biomedicine Research and Development Center, College of Life Science and Technology, Jinan University, Guangzhou, China

**Keywords:** COVID-19, SARS-CoV-2, anti-COVID-19 drugs, remdesivir, lopinavir/ritonavir, chloroquine, plasma therapy, glucocorticoid

## Abstract

The outbreaks of COVID-19 due to SARS-CoV-2 has caused serious physical and psychological damage to global human health. COVID-19 spread rapidly around the world in a short time. Confronted with such a highly infectious respiratory disease, the research and development of anti-COVID-19 drugs became an urgent work due to the lack of specific drugs for the treatment of COVID-19. Nevertheless, several existing drugs are available to relieve the clinical symptoms of COVID-19. We reviewed information on selected anti-SARS-CoV-2 candidate therapeutic agents published until June 2, 2020. We also discussed the strategies of the development of anti-COVID-19 drugs in the future. Our review provides a novel insight into the future development of a safer, efficient, and toxic-less anti-COVID-19 drug.

**Graphical Abstract:**
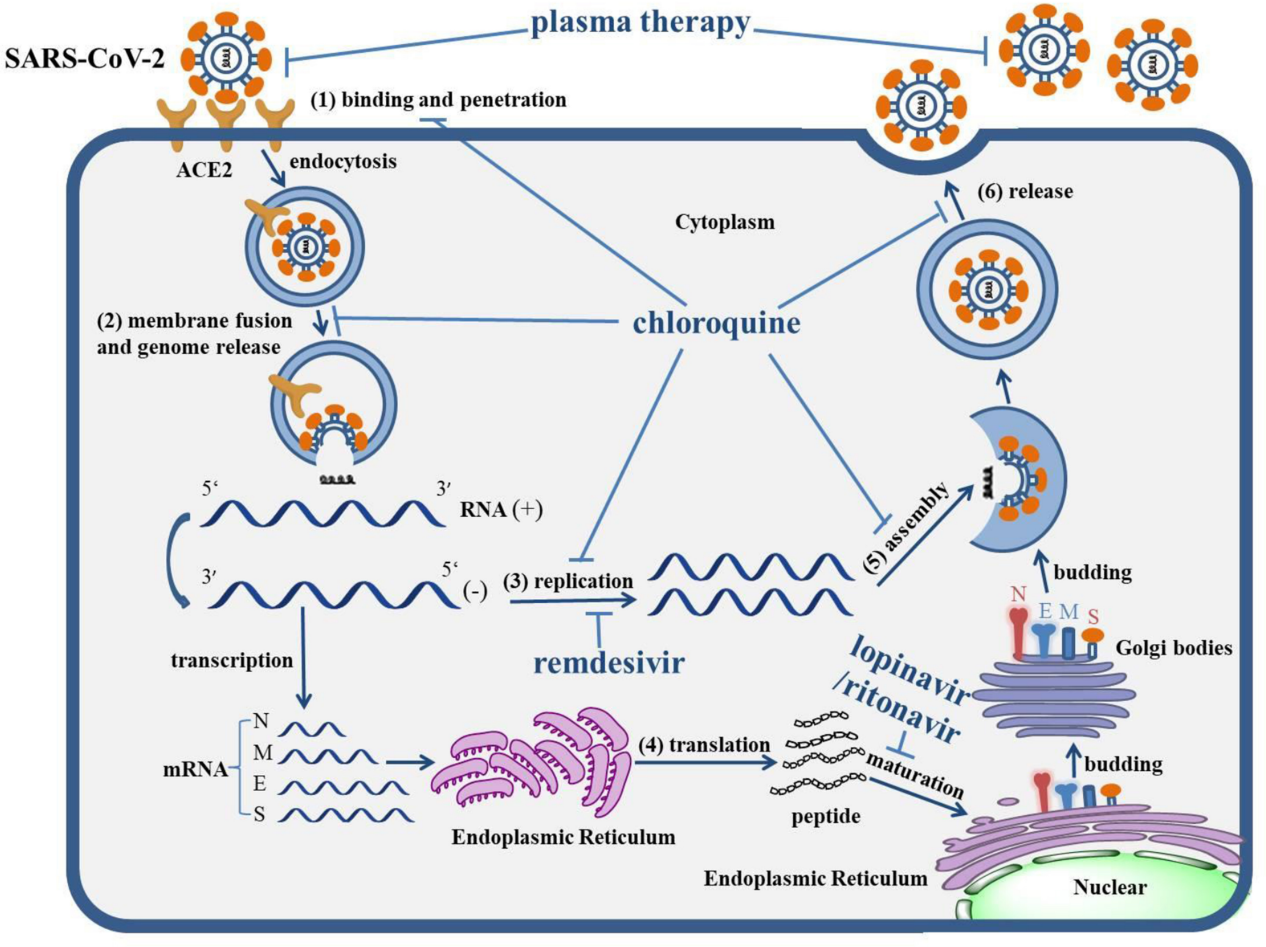
The life cycle of SARS-CoV-2 and the mechanism of actions of anti-COVID-19 drugs. The life cycle of SARS-CoV-2 in host cells includes: (1) Binding and penetration: SARS-CoV-2 binds to the ACE2 receptors on the cell membrane and entry into host cells through endocytosis. (2) Genome release: the genome of SARS-CoV-2 will be released following the process of membrane fusion. (3) Genome replication: the positive (+)-sense genomic RNA directs the synthesis of negative (−)-sense RNA, which can act as the template to synthesize the RNA chain of progeny virus. (4) Protein biosynthesis: negative (−)-sense RNA acts as a template, with mRNAs transcribed to direct the protein biosynthesis of SARS-CoV-2 via the translation process in the cytoplasm. (5) Assembly: the genomic RNA and virion proteins are reassembled to form a mature virion. (6) Release: the progeny viral particles are released through exocytosis. The levels of actions of corresponding drugs were also depicted. Remdesivir can inhibit the replication of the SARS-CoV-2 genome. The combination of lopinavir/ritonavir can block the maturation of protein. Chloroquine virtually interrupts the whole life cycle of SARS-CoV-2. The antibody within plasma can directly neutralize SARS-CoV-2.

## Key Points

- The outbreak of COVID-19 caused by SARS-CoV-2 has presented a challenge to global human health. However, there is no specific drug against COVID-19. It is imperative to summarize the mechanism of action and the therapeutic effect of currently used drugs. Moreover, the side effects of existing drugs against COVID-19 need to be recognized.
- Based on the clinical effects and characteristics of existing drugs, the strategies to develop toxic-less, and more effective anti-COVID-19 drugs were also summarized and posed.

## Introduction

It was reported that a group of patients with pneumonia from an unknown cause were hospitalized at the end of 2019 (1). Most of them had respiratory symptoms, such as fever, cough, muscle soreness, headache, sore throat, chest pain, diarrhea, nausea, and vomiting, and among whom some even developed the complication of acute respiratory distress syndrome (ARDS) (2). This pneumonia was caused by a novel coronavirus termed severe acute respiratory syndrome coronavirus 2 (SARS-CoV-2), a sister of SARS-CoV, as revealed by the subsequent result of gene sequencing (3). The corresponding disease caused by SARS-CoV-2 was named 2019 coronavirus disease (COVID-19) (4). Of note, SARS-CoV-2 exhibited a high level of person-to-person transmission (5), which may be due to the strong affinity with its receptor Angiotensin-Converting Enzyme 2 (ACE2) (6). The ongoing outbreak of COVID-19 had been announced as a global pandemic by the WHO on 11 March, 2020. According to the report from the Center for Systems Science and Engineering at Johns Hopkins University (last updated on 4/26/2020), the global cumulative number of confirmed cases of COVID-19 has reached 2,856,771, with 202,473 deaths (7). Confronted with such a situation, the development of effective drugs for the treatment of COVID-19 has become a crucial and urgent work (8). However, there is currently no specific drugs against SARS-CoV-2, despite some treatments that had been used in the clinical treatment of COVID-19 (9). The mechanisms of actions of these drugs need to be elucidated and discussed. In this review, we summarized and discussed the currently available clinical treatment measures according to their mechanism of action and therapeutic effect (Table 1). The studies presented in this review were obtained from Google Scholar search engines and the PubMed database from searches up to June 2, 2020. Search terms include “COVID-19,” “SARS-CoV-2,” “anti-COVID-19 drugs,” “COVID-19 clinical trials,” and “development strategies of anti-COVID-19 drugs” in abstract, title, and keywords. The strategies of the future development of anti-COVID-19 drugs were also discussed. Our review would be beneficial for the development of more effective and toxic-less anti-COVID-19 drugs.

**Table 1:** Targets, mechanism, usage, limitations, and improvements of the anti-COVID-19 drugs.

**Name**	**Targets**	**Mechanism**	**Dose and usage**	**Limitations**	**Improvements**
Remdesivir	RDRP	Integrated into the RNA chain to inhibit the replication of the viral genome	Intravenous injection, 10-days course, intravenous injection 200 mg for the 1st day and intravenous injection 100 mg for the following days	Hypotension, increased hepatic enzymes, and renal impairment	Unknown
Lopinavir/ritonavir	3CL^pro^	Inactivate the 3CL^pro^ to block the cleaving and maturation of the protein	Peros, the course of treatment should be <10 days, 200 mg/50 mg/capsule, two capsules each time, twice per day	Gastrointestinal effects	Combination with other drugs or film-coated tablet formulation
Chloroquine	Intranuclear body, lysosome, and Golgi body	Increase the pH to block the whole virus life cycle	Peros, the course should be <10 days, <500 mg daily	Arrhythmias, immunosuppression	Dose <500 mg daily
Plasma therapy	SARS-CoV-2	Neutralize the SARS-CoV-2	Intravenously guttae, 200 mL of convalescent plasma with neutralization activity of >1:640	Limited source	Collection, storage, and distribution of plasma is of great importance
Glucocorticoid	Glucocorticoid receptor	Inhibit cytokine storms to prevent tissue and organ damage	Intravenous injection, 3–5 days course, less-than-equal to 1–2 mg/(kg·day) of methylprednisolone	Attenuate the host immunity	Usage and dose should be administered according to the patient's condition

## Remdesivir

The nucleoside analogs are important reagents for combating virus infection (10). As one of the well-characterized adenosine analogs, remdesivir can restrain the proliferation of SARS-CoV, MERS-CoV, and Ebola virus *in vitro* (11). Remdesivir can be integrated into the RNA chain of progeny virus as the substrate of the RNA-dependent RNA polymerase (RdRp), which inhibits the replication of viral genomes and thereby causes the mature termination of the virus (12). It has also been verified that remdesivir can strongly interfere with the accomplishment of the SARS-CoV-2 life cycle in host cells (13). The latest report indicated a clinical improvement of severe COVID-19 patients from multiple countries in 36 of 53 patients (68%) after treatment with remdesivir (14). Due to such an excellent efficacy, remdesivir has entered into multiple clinical trials (15). The results of a randomized, double-blind, placebo-controlled, multicentre trial suggested that whether intravenous remdesivir could decrease the time to clinical improvement in those treated earlier needs to be confirmed by further clinical studies. However, no statistically significant clinical benefits were observed in the remdesivir group compared with the placebo group in this clinical trial (16). Indeed, a patient with COVID-19 successfully recovered after receiving remdesivir intravenously in the United States (17), which further indicates that remdesivir would be rapidly applied as a clinical treatment for COVID-19 in the future. However, remdesivir has been found to cause side effects in the clinic, such as hypotension, increased hepatic enzymes, and renal impairment (14). The mechanism responsible for the side effects of remdesivir is not clear. Further study is needed to address the mechanism of the side effects caused by remdesivir. Collectively, remdesivir is a relatively promising anti-SARS-COV-2 candidate therapeutic agent (18).

## Lopinavir/Ritonavir

Lopinavir is an inhibitor of Human Immunodeficiency Virus 1 (HIV-1) protease (19). The metabolism of lopinavir can be delayed by ritonavir to enhance the anti-HIV-1 effect of lopinavir; therefore, these two drugs are often used in combination (20). The brand name of such a combined drug is Kaletra (21), which displays a broad-spectrum antiviral activity, including on SARS-CoV-2 (22). Mechanism studies suggested that the lopinavir/ritonavir combination may inactivate the 3-chymotrypsin-like cysteine protease (3CL^pro^) that cleaves protein precursors into a variety of active proteins required for the life cycle of SARS-CoV-2 (23). A non-comparative case series of 10 patients suggested that lopinavir may ameliorate the symptoms of COVID-19 (24). After receiving lopinavir/ritonavir with arbidol combination therapy, the negative conversion rate of COVID-19 on the 7 and 14th days was significantly increased (25). Indeed, the viral load of a COVID-19 patient who received lopinavir/ritonavir combination therapy was gradually decreased and even completely cleared within the next few days in Korea (26). A retrospective analysis further supported that lopinavir is an effective drug for the treatment of COVID-19 (27). However, no benefit was observed in COVID-19 patients who were receiving lopinavir/ritonavir combination therapy as revealed by a randomized, controlled, open-label trial (28). Importantly, lopinavir/ritonavir combination (200 mg/50 mg/capsule, two capsules each time, twice per day for adults, the course of treatment should be <10 days) was recommended for the treatment of COVID-19 by the National Health Commission of China. However, the lopinavir/ritonavir combination can induce severe gastrointestinal effects for the treatment of COVID-19, the cause of which remains unknown (28). Of note, the lopinavir/ritonavir combination can be used in combination with other drugs to alleviate adverse reactions, such as probiotics, soluble fiber, and L-Glutamine (GLN) (29). Besides, the film-coated tablet formulation of lopinavir/ritonavir induces fewer gastrointestinal side effects than when used in tablet formulation (30).

## Chloroquine

Chloroquine is a cheap and safe drug that has been used in the clinic for more than 70 years (31). Chloroquine is a first-line drug for the treatment of Plasmodium falciparum infection (32). Importantly, chloroquine also exerts strong antiviral effects (33). Mechanically, chloroquine can increase the pH of the intranuclear body, lysosome, and Golgi body, which jointly prevents virus penetration, genome replication, and assembly of mature viral particles (34). Of note, it was confirmed that chloroquine can suppress the replication of SARS-CoV-2 *in vitro* with an EC_50_ of 1.13 μM (13). Interim analysis of preliminary data from 23 ongoing clinical trials reported in a letter suggested that chloroquine phosphate is superior to the control treatment in inhibiting the exacerbation of COVID-19 pneumonia (35). Hydroxychloroquine, a derivative of chloroquine, also can significantly inhibit the infection of SARS-CoV-2 on VeroE6 cells with weak toxicity (36). An uncontrolled, non-comparative, observational study in a cohort of 80 inpatients reported clinical improvements and rapid fall of viral load after receiving hydroxychloroquine and azithromycin combination therapy (37). However, the administration of hydroxychloroquine alone did not significantly increase the negative conversion rate in COVID-19 patients (38). Indeed, chloroquine phosphate is recommended as an effective treatment by the National Health Commission of the People's Republic of China. There are more than 16 clinical trials aimed at determining the effectiveness of chloroquine in the treatment of COVID-19 (39). If the result of the clinical trial supports the efficacy and safety of chloroquine against COVID-19, chloroquine will become one of the most available drugs for the treatment of COVID-19 (40). Specifically, chloroquine and hydroxyquinoline could impair host immunity by inhibiting toll-like receptor 7 (TLR7) and toll-like receptor 9 (TLR9) signaling due to the increased pH (41). In particular, a high dose of chloroquine and hydroxyquinoline can cause arrhythmias and even death by interfering with the polarization and depolarization of the heart (42). Indeed, it was recommended that <500 mg of chloroquine and hydroxyquinoline is used as a daily dose for adults and it is not advocated for long-term use; if long-term use is needed, the toxic-less hydroxychloroquine should be given priority (43).

## Plasma Therapy

The antibodies against SARS-CoV-2 produced by plasma cells can neutralize the virus to reduce its pathogenicity (44). Scientists have been devoted to the development of antibodies against SARS-CoV-2 since the outbreak of COVID-19 (45). SARS-CoV-2 contains four conserved structural proteins—the spike (*S*) protein, the membrane (*M*) protein, the nucleocapsid (*N*) protein, and the small envelope (*E*) protein (46)—in which the *S* protein shows excellent antigenicity (47). Of note, the SARS-specific human monoclonal antibody CR3022 can bind to the *S* protein of SARS-CoV-2 as determined by enzyme-linked immunosorbent assay (ELISA) and biolayer interferometry binding (BLI) assay, whereas the clinical efficacy of CR3022 needs to be further verified (48). Additionally, it has been reported that the antibodies within convalescent plasma can neutralize SARS-CoV-2 efficiently and rapidly (49). As revealed by a clinical trial with a small sample size, the convalescent plasma may be a potential treatment for COVID-19 patients who were admitted to the intensive care unit (ICU) (50). Similarly, another clinical trial also revealed the remarkable efficacy and feasibility of plasma therapy for the treatment of COVID-19 (51). However, plasma therapy is limited by the shortage of its sources because an ideal therapeutic plasma should be compatible with the recipients (52). Therefore, the collection, storage, and distribution of plasma would be crucial work for the development of plasma therapy (52). Collectively, plasma therapy for the treatment of COVID-19 patients with systemic, severe, and critical conditions requires confirmation in larger studies.

## Glucocorticoid

Glucocorticoid, also known as an adrenocortical hormone, is a steroid hormone secreted by the human adrenal gland (53). As one of the most important physiological hormones, glucocorticoids can regulate the biosynthesis and metabolism of the host (54). Significantly, glucocorticoid also shows a strong activity of anti-inflammation (55). However, the long-term use of glucocorticoids also induces severe side effects, such as the increased risk of osteonecrosis, endocrine disorders, and heart failure (56). During the outbreak of SARS in 2003, the clinical application of glucocorticoids was an inevitable choice for critically ill patients in China (57). Glucocorticoids can inhibit cytokine storms and chemokines caused by SARS-CoV-2 to prevent acute lung injury and acute respiratory distress syndrome (58). Although the clinical evidence does not support glucocorticoid treatment for SARS-CoV-2 infection (59), glucocorticoid can serve as adjuvant therapy for critical patients with COVID-19 (60). The 6th edition of the Diagnosis and treatment plan of Corona Virus Disease 2019 recommended glucocorticoid [≤ 1–2 mg/(kg·day) of methylprednisolone] as an alternative therapy. Of note, glucocorticoid can attenuate the host immunity by inhibiting toll-like receptor 4 (TLR4) signaling and T cell activation, which may cause the secondary infection of other pathogens (61). However, such side effects can be partly restored by the combination of thalidomide and glucocorticoid with a reduced dose of glucocorticoids (62). Therefore, the usage and dose should be administered moderately according to the patient's condition when glucocorticoid is used to relieve inflammation of COVID-19 patients. Collectively, further randomized controlled trials are needed to determine the safety and feasibility of glucocorticoids in relieving inflammatory symptoms of COVID-19 patients (63).

## Other Treatments

In addition to the drugs mentioned above, some drugs with fewer reports showed the activity of anti-SARS-CoV-2. Specifically, cinanserin, another HIV-1 protease inhibitor, may be a potential drug against COVID-19 as indicated by molecular docking and antiviral activity assay (64). It has been recently reported that baricitinib is also a potential option for COVID-19 patients via blocking the ACE2 receptor-mediated endocytosis, although its efficacy remains to be clinically tested (65). Oseltamivir has been widely used for COVID-19 patients although the therapeutic effect on COVID-19 remains to be further explored (66). Some nucleoside analogs, including favipiravir, penciclovir, and ribavirin, can significantly inhibit the proliferation of SARS-CoV-2 *in vitro* (13). Among these, ribavirin can inhibit the replication of both DNA and RNA viruses (67). The combination of ribavirin and lopinavir/ritonavir or interferon can be used to treat COVID-19 as recommended by the 6th edition of Diagnosis and treatment plan of Corona Virus Disease 2019. Arbidol can prevent viral replication by interrupting the virus life cycle and enhancing the immune response (68). The clinical trial of arbidol in the treatment of COVID-19 (NCT04246242) has been registered. Besides, interferon is a broad-spectrum antiviral factor secreted by the host upon the invasion of pathogens. SARS-CoV-2 was more sensitive to interferon than SARS-CoV *in vitro* (69). IFN-α spray is also recommended for the treatment of COVID-19 according to 6th edition of the Diagnosis and treatment plan of Corona Virus Disease 2019. Viral genome editing is also an emerging therapeutic strategy for combating SARS-CoV-2. For example, the CRISPR/Cas13d system delivered by an adeno-associated virus (AAV) serotypes harboring a high affinity with the lungs can accurately excise the genome of SARS-CoV-2 (70). Traditional Chinese medicine is also a tremendous source for anti-COVID-19 drugs (71). For instance, lianhuaqingwen can inhibit the proliferation of SARS-CoV-2 and reduce the induction of inflammatory factors by SARS-CoV-2 (72).

## Conclusions and Future Perspective

The outbreak of COVID-19 raises a serious challenge to the global economy and human health; therefore, the development of an effective treatment for COVID-19 has become an urgent work (73). Indeed, there has been no specific drug against coronavirus since the outbreak of SARS in 2003. The drugs currently used for the treatment of COVID-19 partly refer to those for the treatment of SARS and MERS (74), which are still assessed in clinical trials. We should pay attention to the following aspects in the future development of anti-COVID-19 drugs.

For one thing, the drugs capable of blocking any step of the virus life cycle can be designed as antiviral drugs. For example, camostat mesylate can significantly block the penetration of SARS-CoV-2 by inhibiting the activity of the serine protease TMPRSS2, which is a factor mediating the penetration of SARS-CoV-2 (75). EK1, a pan-coronavirus fusion inhibitor, can interfere with the membrane fusion of SARS-CoV-2 with the host cell by targeting the S protein (76). Similar to remdesivir, sofosbuvir, galidesivir, and tenofovir may also act as the substrate of RdRp to inhibit the SARS-CoV-2 genome replication as revealed by the molecular docking results (77). The 3CL^pro^ enzyme is responsible for cleaving polymeric protein precursors to produce many non-structural proteins that are required for the replication of SARS-CoV-2, which indicates that 3CL^pro^ inhibitors, including celecoxib and alprazolam, can be used to combat COVID-19 (78). Of note, based on targeting the ACE2 receptor, a previous study had discovered several potential anti-COVID-19 drugs by using computational methods, such as xanthones and hesperidin (79). Indeed, the lack of specific drugs for combating SARS-CoV-2 was largely due to the incomprehensive recognition of the mechanism of SARS-CoV-2 infection in host cells (80). Therefore, future work should focus on exploring the life cycle of SARS-CoV-2 in human cells and the detailed mechanism of the pathogenesis of SARS-CoV-2.

For another, the alleviation of host inflammation is an essential and urgent work for COVID-19 patients with cytokine storm (81). Of note, in addition to glucocorticoids mentioned above, there are other agents with an anti-inflammatory effect, such as tocilizumab and jakotinib, an interleukin-6-receptor antagonist and Janus kinase (JAK) inhibitor, respectively (82). Further, screening from FDA-approved drugs based on computational methods would be an ideal strategy to ensure the efficiency of anti-COVID-19 drugs development (83). Indeed, the vaccine is crucial for the prevention and control of COVID-19. Scientists need to have a better understanding of the interaction between SARS-CoV-2 and the immune system. The S protein is an ideal antigen for the development of vaccines due to its high affinity with the ACE2 receptor (84). In particular, the receptor-binding domain (RBD) element of the *S* protein may be applied to vaccine development (85).

## Author Contributions

LH participated in data collection, data analysis, drafting, and editing of the manuscript. YilW contributed to the critical review and revision of the manuscript. All authors reviewed, supported the final manuscript, and agreed to this publication.

## Conflict of Interest

The authors declare that the research was conducted in the absence of any commercial or financial relationships that could be construed as a potential conflict of interest.

## Abbreviations

ARDS: acute respiratory distress syndrome
SARS-CoV-2: severe acute respiratory syndrome coronavirus 2
COVID-19: 2019 coronavirus disease
ACE2: Angiotensin-Converting Enzyme 2
RdRp: RNA-dependent RNA polymerase
HIV-1: Human Immunodeficiency Virus 1
3CL^pro^: 3-chymotrypsin-like cysteine protease
GLN: L-Glutamine
TLR7: toll-like receptor 7
TLR9: toll-like receptor 9
S: spike
M: membrane
N: nucleocapsid
E: envelope
ELISA: enzyme-linked immunosorbent assay
BLI: biolayer interferometry binding
ICU: intensive care unit
TLR4: toll-like receptor 4
AAV: adeno-associated virus
JAK: Janus kinase
RBD: receptor-binding domain.
